# Fatty Acid Reference Intervals in Red Blood Cells among Pregnant Women in Norway–Cross Sectional Data from the ‘Little in Norway’ Cohort

**DOI:** 10.3390/nu12102950

**Published:** 2020-09-25

**Authors:** Pedro Araujo, Marian Kjellevold, Ive Nerhus, Lisbeth Dahl, Inger Aakre, Vibeke Moe, Lars Smith, Maria Wik Markhus

**Affiliations:** 1 Institute of Marine Research, 1870 Nordnes, N-5817 Bergen, Norway; i.nerhus@gmail.com (I.N.); Lisbeth.Dahl@hi.no (L.D.); Inger.Aakre@hi.no (I.A.); Maria.Wik.Markhus@hi.no (M.W.M.); 2 Department of Psychology, Faculty of Social Sciences, University of Oslo, 0317 Oslo, Norway; vibeke.moe@psykologi.uio.no (V.M.); lrssmth@gmail.com (L.S.)

**Keywords:** fatty acid status, pregnancy, nutrition, biomarker, seafood intake, ω-3 supplement

## Abstract

There is a growing interest in determining fatty acid reference intervals from pregnancy cohort, especially considering the lack of reference values for pregnant women in the literature and the generalized misconception of equating reference intervals for nonpregnant women as equivalent to pregnant women. Seafood and supplements are important dietary sources for the omega-3 long-chain polyunsaturated fatty acids (ω-3 LCPUFA), such as eicosapentaenoic acid (EPA, 20:5ω-3), docosapentaenoic acid (DPA, 22:55ω-3), and docosahexaenoic acid (DHA, 22:6ω-3). Sufficient intake of EPA and DHA is vital during pregnancy for the development of the fetus, as well as for maintaining adequate levels for the mother. This study describes the fatty acid status and suggests reference values and cut-offs for fatty acids in red blood cells (RBC) from pregnant women (*n* = 247). An electronic food frequency questionnaire (e-FFQ) mapped the dietary habits of the participants, and gas chromatography was used to determine the fatty acid levels in RBC. The association between e-FFQ variables and fatty acid concentrations was established using a principal component analysis (PCA). Twenty-nine-point-one percent (29.1%) of the participants reported eating seafood as dinner according to the Norwegian recommendations, and they added in their diet as well a high percentage (76.9%) intake of ω-3 supplements. The concentration levels of fatty acids in RBC were in agreement with those reported in similar populations from different countries. The reference interval 2.5/97.5 percentiles for EPA, DPA, DHA were 0.23/2.12, 0.56/2.80, 3.76/10.12 in relative concentration units (%), and 5.99/51.25, 11.08/61.97, 64.25/218.08 in absolute concentration units (µg/g), respectively. The number of participants and their selection from all over Norway vouch for the representativeness of the study and the validity of the proposed reference values, and therefore, the study may be a useful tool when studying associations between fatty acid status and health outcome in future studies. To the best of our knowledge, this is the first PCA study reporting a direct association between ω-3 LCPUFA and intake of seafood and ω-3 supplements in a pregnancy cohort.

## 1. Introduction

Nutrient deficiencies may lead to undesirable health outcomes. Pregnant women are considered vulnerable, as the mother is the sole provider of nutrients for the fetus [1,2,3]. During pregnancy and lactation, the maternal fatty acid status declines [4,5], which may lead to a suboptimal supply for the fetus, principally in cases where the dietary intake of these fatty acids is low or absent. In addition, fatty acids are released from maternal adipose tissue stores to the fetus, especially docosahexaenoic acid (DHA, 22:6ω-3), and marginally change blood levels [3,6]. The rapid growth of the fetal brain during pregnancy and the first two years of childhood demand adequate levels of nutrients, such as the omega-3 long-chain polyunsaturated fatty acids (ω-3 LCPUFA), eicosapentaenoic acid (EPA, 20:5ω-3), and DHA. Experimental evidence suggests that DHA is the major structural and functional fatty acid in the central nervous system [5,7]. Consequently, the maintenance of maternal fatty acid supply is crucial.

Norway recommends a daily intake of 200 mg DHA for pregnant women [8]. Aquatic foods and ω-3 supplements are the main dietary sources of EPA and DHA [9]. Pregnant women are advised to follow the general dietary recommendations, which is to consume 300–450 g of fish per week, corresponding to fish or fish products for dinner 2–3 times per week, of which a minimum of 200 g should be fatty fish. There is inconsistency regarding the effects of DHA supplementation during pregnancy and in the early phase of infant cognitive development. Some research suggests a beneficial effect of DHA supplementation during pregnancy and/or lactation on mental development and on long-term cognition [10]. However, the evidence on cognitive development is inconclusive [11,12,13,14,15]. Recent studies have also concluded that low levels of ω-3 LCPUFA in the blood are a risk factor for early preterm birth and that an increased intake of ω-3 LCPUFA (via fish or supplements) is advisable [6,16]. Some studies suggested that pregnant and lactating women should consume 225–350 g (8–12 oz.) per week (~250–375 mg/day of EPA and DHA) of a variety of seafood [17]. However, a study on DHA and the increased risk for early preterm birth recommends a range of 600–800 mg/day of DHA for women with levels of DHA in red blood cells (RBC) lower than 5% [6]. Some authors who support the supplementation of ω-3 LCPUFA as an effective strategy for reducing preterm birth advise that a follow-up of completed trials is needed to assess long-term outcomes [18]. Lands and collaborators emphasize that careful handling of data on fatty acid composition is needed when interpreting evidence of dietary fatty acids on health outcomes [19].

Determination of fatty acid levels in RBC is a well-known approach for assessing fatty acid status as it reflects the last 30–60 days of intake [20]. EPA and DHA, accompanied by some other fatty acids, for example, short-chain fatty acids present in milk, are indirect biomarkers of specific foods as these foods are the primary dietary source of the respective fatty acids [21].

Reference intervals provide information on specific biomarkers in population-based cohort studies and offer a clear understanding of the initial status, as well as provide the basis for comparison over time. Most laboratories and scientific reference tables offer information derived from healthy nonpregnant women, but lack reference intervals for pregnant women. During pregnancy, there are changes in many biological markers, and therefore, reliable reference values derived from a healthy pregnant population are of importance for correct clinical decisions. Without adequate reference intervals, there is an increased risk of missing important changes, due to pathological conditions and to erroneously interpretation of normal changes as pathological events [22]. Hence, reference intervals are the most widely used tool for medical decision-making, therapeutic management decisions, and other physiological assessments [23,24]. The present study aims at suggesting reference intervals and cut-offs for fatty acids in maternal RBC on a sufficiently large healthy population that can be used in future studies to identify women who are at risk of adverse health outcomes as a result of under or overexposure to fatty acids. In addition, the relationship between the intake of seafood and ω-3 LCPUFA, generally characterized as poor in many pregnancy cohort studies [25,26], is thoroughly investigated using a principal component analysis.

## 2. Materials and Methods

### 2.1. Study Design

The present research is based on data from the national Little in Norway (LiN) cohort project (ISRCTN registry number 66710572) that is a cross-disciplinary prospective longitudinal study starting in pregnancy. The overall study design for the LiN-cohort has been described in more detail elsewhere [27]. The LiN-cohort included nine health care centers from northern, mid, western, and eastern Norway (Table 1).

**Figure 1:**
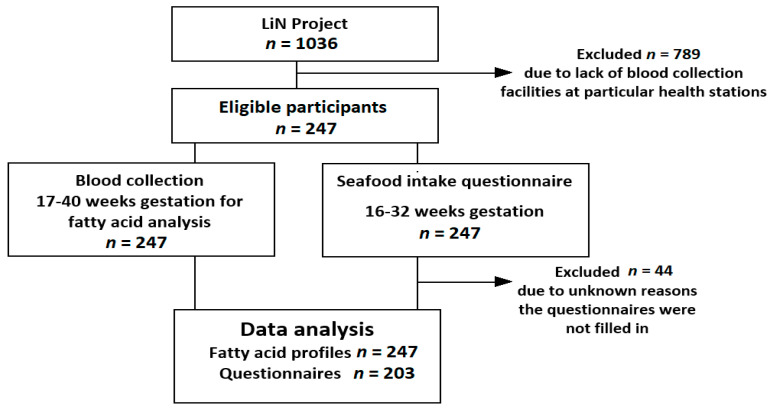
Flow chart of the study population, including reasons behind patient exclusion and refusals.

The study was conducted from September 2011 to October 2012 according to the guidelines laid down in the Declaration of Helsinki. The procedures involving human subjects were approved by the Regional Committees for Medical and Health Research Ethics in Norway (REK 2011/560). Informed written consent was obtained from all subjects participating in the study. The flow of participants and data relevant for this research is outlined in Figure 1.

### 2.2. Population

**Figure 2:**
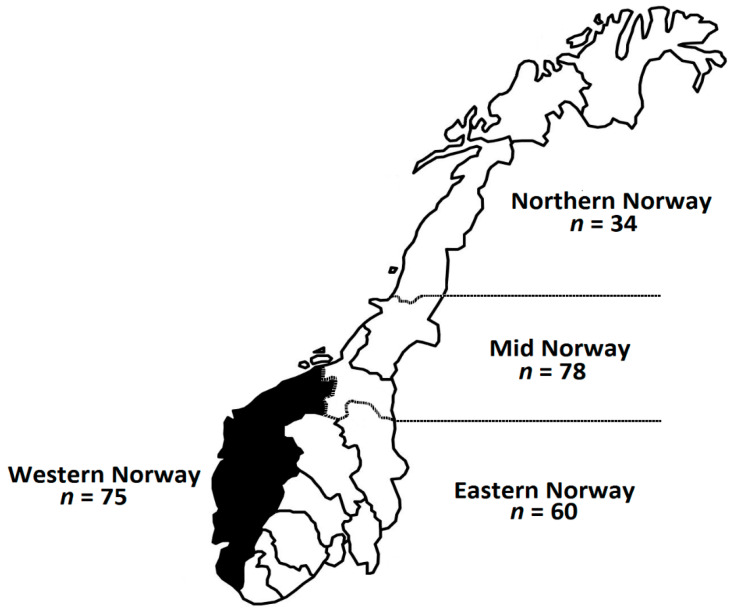
Norwegian map showing the geographical location and distribution of the participants (*n* = 247).

Pregnant women (*n* = 247), at different gestational periods and from different geographical regions in Norway (Figure 2), were recruited and their blood collected at the first prenatal appointment in the health centers. The characteristics of the population, including age, gestational weeks, and demographic information is presented in Table 1.

### 2.3. Dietary Assessment

The validated electronic semi-quantitative food frequency questionnaire (e-FFQ) [28] was implemented on 203 out of the 247 participants to determine the dietary intake of seafood between gestational weeks 16 and 32. The e-FFQ considers questions, such as “How often have you consumed fish, fish products or other seafood as lunch, spread or snack meal during the last three months?” and also a question regarding intake of ω-3 supplements, with the alternatives “yes” and “no”. The e-FFQ was designed to capture the whole seafood diet, including seafood from all meals during the day [28]. Educational level, demographic information, and tobacco use questions are also included in the e-FFQ. The participants were anonymized by giving a unique ID number and corresponding password for entering the electronic questionnaire. It is important to mention that before starting the LiN project, all the available brands of omega-3 supplements in Norway were analyzed, and the results were published elsewhere [29]. The fatty acid composition in mg/capsule of the different brands of ω-3 supplements that were consumed by the participants is reported in Table S1. The declared content of a capsule was always 1 g of oil. The minimum/maximum levels of EPA, DPA, DHA, and EPA+DHA in mg/capsule were 174.55/282.65, 25.05/41.45 167.60/190.75, and 349.65/457.00, respectively (Table S1).

### 2.4. RBC Collection

The sample collection procedure has been described elsewhere [4]. Briefly, non-fasting venous blood samples from the participants were collected by venepuncture in 4 mL BD Vacutainer K_2_EDTA (7.2 mg) vials (Becton, Dickinson and Company, Franklin Lakes, USA) at the first prenatal appointment. The vials were centrifuged (1000–1300× *g*, 20 °C, 10 min) within 30 min. The RBC were adequately separated from plasma and buffy coat to ensure a clean RBC fraction. The samples were stored at the sites of the collection at −18 °C for up to a maximum of four weeks, and thereafter shipped to the Institute of Marine Research (IMR) in Bergen, Western Norway, for further storage at −80 °C prior to analysis. Regarding the stability of the fatty acids in the RBC samples, some studies recommend temperatures between 1 °C and 6 °C to preserve RBC quality for up to 42 days [30]. In addition, a recent pilot biobank study, at IMR, demonstrated that fatty acid profiles from RBC, with or without antioxidants, remain stable for up to 13 weeks at −20 °C and −80 °C [31].

### 2.5. Fatty Acids

The preparation of the fatty acid methyl ester (FAME) is an accredited method granted by the Norwegian Accreditation Authority and published elsewhere [32]. Briefly, 50 μL of the RBC sample was mixed with 2 mL BF_3_ in methanol, and 5 μg of 19:0 internal standard. The mixture was heated at 100 °C for 1 h and cooled until it reached room temperature. Aliquots of 1 mL of hexane and 2 mL of H_2_O were added, vortex-mixed for 15 s, placed in a centrifuge at 1620× *g* for 2 min, and the hexane phase (containing the FAME) was collected, evaporated under nitrogen, dissolved in hexane, and submitted to gas chromatography analysis at IMR on a Perkin-Elmer AutoSystem XL gas chromatograph (Perkin-Elmer, Norwalk, CT, USA) equipped with a liquid autosampler and a flame ionization detector. The FAME samples were analyzed on a CP-Sil 88 capillary column (50 m × 0.32 mm I.D. 0.2 µm film thickness, Varian, Courtaboeuf, France). Data collection was performed by the Perkin-Elmer TotalChrom Data System software version 6.3 (Perkin-Elmer, Somerset, MA, USA). The temperature program was as follows: The oven temperature was held at 60 °C for 1 min, ramped to 160 °C at 25 °C/min, held at 160 °C for 28 min, ramped to 190 °C at 25 °C/min, held at 190 °C for 17 min, ramped to 220 °C at 25 °C/min and finally held at 220 °C for 10 min. The direct on-column injection was used. The injector port temperature was ramped instantaneously from 50 to 250 °C, and the detector temperature was 250 °C. The carrier gas was ultra-pure helium at a pressure of 82 Kpa. The analysis time was 60 min. This time interval was sufficient to detect FAME with chains from 10 to 24 carbons in length. The FAME peaks were identified by comparing their retention times with the retention times of highly purified FAME standards. The fatty acid results were expressed as relative (%) and absolute (mg/g RBC wet weight) units. The omega-3-index was calculated as the sum of EPA and DHA in relative units (Σ(%EPA + %DHA) [33].

### 2.6. Statistics

An Excel-based platform (Table S2) was developed for the automatic analysis of the chromatographic data. The Excel-based platform consists of three workbooks: (1) Data entry, where a maximum of five fatty acid concentration profiles can be entered; (2) FA distribution per station, where the distributions of the different fatty acids at the different health care stations are displayed automatically; (3) total FA distribution, to visualize automatically whether the total number of measured concentrations (*n* = 247) for specific fatty acids are normally distributed. The percentiles of the fatty acids were derived from the normal distribution. After transforming the e-FFQ nominal variables into numerical values, they were submitted to principal component analysis (PCA) along with the chromatographic data to detect meaningful relationships between the different fatty acids and the intake of seafood and ω-3 supplements. Statgraphics Centurion XVI (Version 16.1.11, StatPoint Technologies, Inc., Warrenton, VA, USA) was used for data analysis.

## 3. Results

### 3.1. Characterization of Study Population

Demographic information of the population, such as age, gestation period, body mass index (BMI), education, marital status, smoking habits (Table 1), and geographical region (Figure 2), are described. The different characteristics were estimated from the total number of participants (*n* = 247), except the BMI values that were estimated from 202 participants and categorized as underweight (3.5%), normal weight (68.8%), and overweight (27.7%). All the participants attended university or university college, and the majority of them (86.2%) were located in geographical regions under Northern Norway (13.8%) (Table 1, Figure 2).

### 3.2. Seafood Intake

The results of the e-FFQ (Table 2) revealed that 76.4% (47.3 + 29.1) of the pregnant women consumed seafood as dinner with a frequency of 1–3 times/week. However, only 29.1% of the participants were following the Norwegian recommendations of seafood intake as dinner 2–3 times/week (Table 2). A percentage of 4.4% of the population reported a frequency intake of seafood as dinner lower than once a month, and from this group of participants, only 2.5% reported consuming ω-3 supplements. For the intake of seafood as spread or snack, similar frequencies were reported for 1–3 times per month (27.6%) and 1–2 times per week (29.1%), and they were ascribed to a relatively high intake of bread and spread in Norway. These particular frequency groups, reported the highest intake of ω-3 supplements, 20.2% (1–3 times per month) and 24.6% (1–2 times per week).

### 3.3. Fatty Acid Status

**Figure 3:**
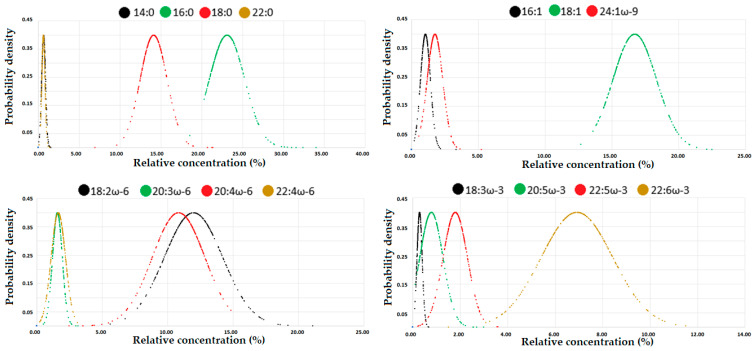
Normal distribution of the different groups of fatty acids (unsaturated, monounsaturated, and polyunsaturated). The graphs can be generated automatically (for % or mg/g) by copy-pasting the experimental results in Table S3 into the provided Excel-based platform in Table S2.

A total of 247 fatty acid concentration profiles were estimated from seven health stations (two out of the total nine health stations lacked facilities for blood collection and sample preservation) and reported in both relative (%) and absolute (mg/g) units (Table S3). The relative concentrations of the fatty acids (14:0, 16:0, 18:0, 22:0, 16:1, 18:1, 24.1ω-9, 18:2ω-6, 20:3ω-6, 20:4ω-6, 22:4ω-6, 18:3ω-3, 20:5ω-3, 22:5ω-3 and 22:6ω-3) at the different health stations were normally distributed. After demonstrating data normality at the different stations, the concentrations of the different fatty acids (unsaturated, monounsaturated, and polyunsaturated) were added together, and graphs of the probability density function against the concentration of fatty acid in the relative unit (%) were plotted (Figure 3) and used for computing the corresponding percentiles (Table 3). Although the distributions of the fatty acids in mg/g units are not shown, they were also normally distributed. The reader can automatically generate the normal distributions (for % or mg/g) by copy-pasting the experimental results in Table S3 into the provided calculation platform in Table S2.

The most concentrated saturated, monounsaturated, ω-6 polyunsaturated fatty acids (PUFA) and ω-3 PUFA in Table 3 were 16:0, 18:1, 18:2ω-6, and 22:6ω-3, respectively. The computed median/average ratios for these major fatty acids were 0.98 (22.7/23.1), 0.99 (16.6/16.7), 0.96 (11.5/12.0), and 1.00 (6.9/6.9) in % units and 0.98 (500.9/509.4), 0.97 (358.6/371.5), 0.95 (255.7/268.6), and 1.00 (153.4/152.7) in µg/g units. Similarly, the rest of the fatty acids exhibited mean/average ratios close to 1.00, indicating that the graph’s probability density versus concentration (Figure 3) provides a good approximation of the sampling distribution of the fatty acid of interest. A comparison of the results in Table 3 with those reported in similar studies was performed, and presented in Table 4.

**Figure 4:**
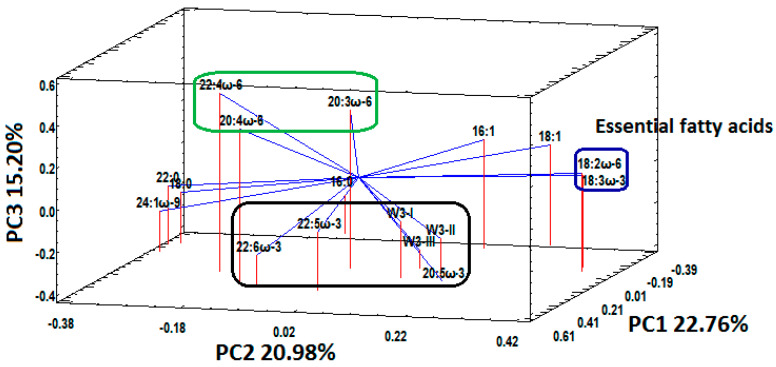
Principal components 1, 2 and 3 (PC1, PC2 and PC3, respectively) to study the correlation between selected fatty acids in maternal red blood cells and electronic food frequency questionnaire (e-FFQ) variables (WI = seafood as dinner, WII = seafood as spread or snack, WIII = ω-3 supplements) as. There is an association between 20:5ω-3 (EPA), 22:5ω-3 (DPA), 22:6ω-3 (DHA) and WI, WII, WIII (black frame), while their ω-6 counterparts (green frame) and essential fatty acids (blue frame) do not correlate with the e-FFQ variables.

The PCA of the e-FFQ and fatty acid data was performed after transforming the e-FFQ nominal data into numerical variables. The transformation consisted of assigning scores of 1 (lowest frequency), 5 or 6 (highest frequency) to the seafood frequency, and scores of 0 (negative answer) or 1 (affirmative answer) to the intake of ω-3 supplements (Table 2). The PCA revealed a positive correlation between EPA (20:5ω-3), docosapentaenoic acid (DPA, 22:5ω-3), DHA (22:6ω-3), the intake of seafood (designated as WI and WII variables in Figure 4) and the intake of ω-3 supplements (designated as WIII variable in Figure 4). These variables (EPA, DPA, DHA, WI, WII, WIII) are close to each other (framed in black in Figure 4) and display negative PC3 values. In contrast, the ω-6 PUFA, more specifically 20:3ω-6, 20:4ω-6, and 22:6ω-3 (framed in green in Figure 4) do not correlate with the e-FFQ variables and display positive PC3 values, which in turn discriminates the ω-3 PUFA. Linoleic acid (LA, 18:2ω-6) and alpha linolenic acid (ALA, 18:3ω-3) emerge as a cluster (framed in blue in Figure 4) and do not exhibit any association with the ω-6 and ω-3 PUFA or any of the studied e-FFQ variables. The remaining fatty acids were independent of the intake of seafood or ω-3 supplements, as observed in Figure 4.

## 4. Discussion

The applied e-FFQ was not focused on ω-3 fatty acids originating from plants, but from the habitual intake of seafood (fish and shellfish) and the use of dietary supplements, because the endogenous metabolization of ALA (18:3 n-3) from plants to ω-3 PUFA (e.g., EPA, DPA, and DHA) is minimal. Furthermore, the e-FFQ considered different forms of seafood individually. For example, the indexes for dinners were grouped into five categories comprising dinner items of oily fish, lean fish, shellfish, processed fish, and freshwater fish. Additionally, freshwater fish consumption was divided into two separate questions, frequency of perch/pike (lean fish) and frequency of char/whitefish (oily fish) [28].

The e-FFQ indicated that 29.1% of the participants reported an intake of fish for dinner that was in accordance with dietary guidelines from the Norwegian Directorate of Health (Table 2). However, a high percentage of participants from all the assessed groups (under and over seafood as dinner 2–3 times/week) reported the intake of ω-3 supplements. In addition, it was remarkable that the intake of ω-3 supplements was almost identical (around 77%) for all the observed groups, 1–3 times per month (27/35 × 100 = 77.14%), one time per week (74/96 × 100 = 77.08%) and 2–3 times per week (46/59 × 100 = 77.97%), as shown in Table 2. The high intake of omega-3 supplements in this particular cohort of Norway is in accordance with global awareness towards the beneficial effects of these dietary products as they improve the levels of omega-3 PUFA by covering dietary seafood shortfalls, particularly for those who dislike the taste or smell of fish.

The observed frequencies for gestational weeks 16 and 32 of 68.97, 29.06 and 76.85% for the categories seafood intake under dietary guidelines (*n* = 140), 2–3 times/week (*n* = 59) and intake of ω-3 supplements (*n* = 156), respectively (Table 2) are consistent with those reported for gestational week 22 and 32 by The Norwegian Mother and Child Cohort Study (*n* = 67007) of 60.06, 23.47 and 63.95 for the categories seafood intake under 2–3 servings/week (*n* = 40244), seafood intake of 2–3 servings/week (*n* = 15724) and intake of ω-3 supplements (*n* = 428852), respectively [34]. In addition, the observed 29.06% frequency (for those Norwegian pregnant women (30.1 ± 4.6 years) in accord with the national dietary guidelines), is in close agreement with the latest national dietary survey conducted among adults in Norway (2010–2011) where women in the age group 30–39 reported a frequency of 21% for the intake of seafood for dinner three times per week or more [35]. The agreement with previous studies confirms the robustness of the semi-quantitative e-FFQ to assess the dietary intake of seafood and ω-3 supplements.

The PCA plot (Figure 4) detected a correlation between the ω-3 PUFA and the e-FFQ variables, and it discriminated the ω-6 and ω-3 PUFA into three clusters that can be intuitively explained, as follow: The concentration levels of 20:3ω-6, 20:4ω-6, and 22:4ω-6 (inside the green frame in Figure 4) and 20:5ω-3, 22:5ω-3 and 22:6ω-3 (inside the black frame in Figure 4) reflect both endogenous (de novo lipogenesis) and exogenous (dietary intake) sources; whereas, the concentration levels of essential fatty acids, such as 18:2ω-6 and 18:3ω-3 (inside the blue frame in Figure 4), exclusively reflect the dietary intake of the participants. In addition, Figure 4 reveals that neither 18:2ω-6 nor 18:3ω-3 are correlated with any of the e-FFQ variables.

The associations between qualitative variables (e.g., frequency of consumption of fish, BMI, ethnicity, etc.) and fatty acids in plasma from pregnant adolescents (14–18 years old) by using PCA has been published elsewhere [36]. Although this particular study did not discuss in detail the PCA results, an analysis of its reported PC1 and PC2 loadings revealed that the association 20:4ω-6/fish was stronger than the association ω-3 PUFA/fish (e.g., 18:3ω-3, EPA, DPA); and also the lack of correlation between essential fatty acids (e.g., 18:3ω-3, 18:2ω-6) which should exclusively reflect the dietary intake. In general, studies on the association between food intake variables from FFQ and fatty acids from pregnant women, by using techniques different to PCA, have consistently demonstrated poor correlations between dietary fatty acid intake and blood levels [25,26]. The present pregnant cohort study is the first to report a clear association between e-FFQ variables and fatty acids in RBC from the pregnant cohort by using PCA.

Except for 18:2ω-6, the sequence of most concentrated fatty acids reported in the present study (16:0, 18:1, 18:2ω-6, 22:6ω-3) has been also observed in studies with pregnant women from Belgium [37], Netherlands [38], Germany [39], and Japan [40,41]. In these countries, the major ω-6 PUFA was 20:4ω-6, and its level was consistently higher than 18:2ω-6 by 69.6, 2.8, 1.0, and 28.2% (average from References [40,41]), respectively; whereas, in the present study 20:4ω-6 was lower than 18:2ω-6 by 9.5%. Possible explanations behind the observed reduction in the present study might be the high intake of ω-3 supplements (76.9%) compared to the studies from Belgium (24.6%), Netherlands (14.3%), Germany (20%), and Japan (2.2% in Reference [41]). In addition, an analysis of the estimated global seafood consumption per country [42] by the time these specific studies were performed indicated that Norway had the highest seafood consumption per capita (52.9 Kg in 2012) compared to Belgium (23.8 Kg in 2016), Netherlands (22.11 Kg in 2000), Germany (14.3 Kg in 2011) and Japan (48.6 Kg in 2013).

In the present study, the ω-3 PUFA sequence ranked from lowest to highest concentration was 18:3ω-3, 20:5ω-3, 22:5ω-3, and 22:6ω-3. This specific sequence is in agreement with similar studies from the Netherlands [38], Germany [39], and Japan [41]. Other studies from Japan [40], Belgium [37], and Iceland [43] have not reported the concentration levels of 18:3ω-3 or 22:5ω-3. However, in these studies, the declared ω-3 PUFA followed the aforementioned order. 

In general, the range of concentrations for selected fatty acids in RBC from pregnant women in Table 3 is in agreement with reported median or average values in similar studies from different countries, as indicated in Table 4 in green color. However, in some countries, the levels of particular fatty acids were distinct from the 2.5 or 97.5 percentiles of the present study, as indicated in Table 4 in yellow and red colors, respectively. The reasons behind the observed discrepancies are beyond the scope of the present article.

Some studies have indicated that values ≥8% or <5% are associated with the lowest risk for cardiovascular events [44] or the highest risk of depressive episodes [45], respectively. Despite these observations, an optimal range of omega-3 index for pregnant women has not been defined yet. A recent study has indicated that no human being has an omega-3 index <2% [44]. Contrary to this observation, in the present study that involved only healthy pregnant women, a participant (hereinafter referred to as p#159) with an omega-3 index of 1.93% was recorded. The relative concentrations of EPA (0.43%) and DHA (1.50%) for p#159 were allocated inside the range and under the lowest percentiles for these fatty acids (Table 3). In addition, p#159 exhibited the largest DPA concentration level (3.59%). A close inspection of the same fatty acids in µg/g units for p#159 revealed that EPA, DHA, and DPA were allocated in the 55, 35, and 55 percentiles, respectively, and consequently, the values in µg/g units are inside the range of the studied population. It is equally important to mention that the ω-6/ω-3 index is another key player in epidemiological studies that are generally associated with depression [45] and cardiovascular events [46]. Some studies have indicated that ω-6/ω-3 >9 is associated with postpartum depression [47]; whereas, an ω-6/ω-3 around 4 exerts cardioprotective effects [46]. Experimental evidence suggests that the optimum ω-6/ω-3 ratio must be kept around 4 and 5 and should not exceed 10 [48]. The computed ω-6/ω-3 ratio for p#159 was 3.84 (95 percentile in Table 3), and it can be regarded as optimum. The previous observations about the different indexes and measurement units, do not try to draw general conclusions based on the results of just one participant, but to highlight the importance of a comprehensive evaluation of the implications in human health of the different indexes and their corresponding threshold not only from the perspective of relative units (%), but also absolute units (mg/g). In addition, it is important to highlight that published randomized trials have not provided conclusive evidence yet about the effect of ω-3/ω-6 PUFA on postpartum depression.

In the present research, 42% of the pregnant women had an omega-3 index above 8%. It was mentioned that this index plays a pathophysiologic role in depressive symptoms [45,49,50]. The International Society for Nutritional Psychiatry Research Practice Guidelines for ω-3 fatty acids has recently recommended therapeutic dosages of pure EPA or a combination of EPA and DHA (with net EPA starting from at least 1 up to 2 g/day) for at least eight weeks as a potential treatment for major depressive disorders [51]. We have previously shown that low omega-3 index in pregnancy is a possible risk factor for postpartum depression [52], with a cut-off at 4%. This cut-off is similar to the 2.5 percentile in Table 3 and in accordance with the cut-off for those at high risk of developing coronary heart disease [53]. Thus, the suggested reference values and omega-3 index cut-off could help to identify women who might benefit from increasing the dietary intake of EPA and DHA, like seafood and supplements that are important dietary sources of these long-chain PUFA, and hence, will influence their nutritional status. It must be mentioned that there are no specific recommendations on the intake of EPA or DHA for the general population, including prenatal women, in Norway [54].

Cohort studies for establishing national reference intervals for fatty acids in RBC of pregnant women are largely dependent, among other things, on the number of participants, the number of health stations, the geographical distribution of the health stations along with their inherent infrastructure for collecting and preserving samples long-term at appropriate temperatures. For instance, fatty acids in RBC are susceptible to degradation and remain stable for 42 or 91 days at 1 °C or −20°C, respectively [30,31]. Failure to comply with these requirements might be regarded as a drawback. Some of the apparent limitations of the present study are the lack of blood collection/preservation facilities (namely seven well-equipped facilities). However, most of the studies in Table 4 were performed in one specific geographical region by using just one blood collection facility. In some cases, the selected geographical regions represented a very low percentage of the total female population of the country in question. For example, the studies from Belgium [37], the Netherlands [38], and Germany [39] represented ~1.71, ~0.71%, and ~0.13% of the total female population, respectively. Moreover, the studies from Iceland [43] and Japan [40,41] constituted approximately 33.73 and 18.23% of the total female population, respectively, they were carried out in specific regions (Reykjavik and the Miyagi Prefecture), and they do not contain all the important characteristics of the country population from which they were drawn. The present study collected samples from the main geographical regions of Norway, which account for a ~91.5% of the targeted population. In addition, the present study with seven collection facilities has a higher level of enrolment per thousand pregnant women than Japan with 15 collection facilities, namely, 4.16‰ and 1.63‰ by the time these specific studies were performed, respectively. The expression *n* = N/[1 + N(e/100)^2^] (aka Slovin formula) [55], that is generally considered to estimate the sample size (n) given the population size (N) and a percentage of margin error (e) was used to judge whether *n* = 247 was an appropriate sample size. By the time the samples were collected (2011–2012), the parameter N was estimated using the Statistics Bureau of Norway’s records of the average number of births (59410 ± 10) between 2011–2012 [56], while the parameter e was set at 7.5% (half the maximum margin of error of 15% proposed by IUPAC for monitoring fatty acid concentrations by gas chromatography [57]). A minimum value of *n* = 177 was calculated by introducing the aforementioned parameters in the Slovin expression, which in turn concluded that the sample size of the present research (*n* = 247) was sufficient to determine reliable reference intervals for fatty acids in maternal RBC. An important feature of a selected sample size should be its ability to make projections or generalizations regarding an entire population. The information in Table 1 and Figure 2 indicates that pregnant women were recruited from all over the Norwegian territory, which emphasizes the strength and representativeness of the sample size, and consequently, the validity of the proposed reference values in the present study. The previous observations indicate that there is not any suspicion of misrepresentation of the population of interest in the present study.

## 5. Conclusions

Reference intervals and cut-offs for fatty acids in RBC from a pregnancy cohort from all over Norway and in agreement with those reported in other countries were established. A direct association between ω-3 LCPUFA (EPA, DPA, DHA, but not ALA) in maternal RBC and the intake of seafood and ω-3 supplements was found. The findings from the e-FFQ were in accordance with national surveys and highlighted the awareness of the participants about the importance of dietary ω-3 in maternal health. Given the importance of seafood and ω-3 supplements during pregnancy, further studies are warranted to investigate comprehensively the impact on the health of the various indexes (e.g., omega-3 index, ω6/ω3) associated with fatty acid status and by using relative and absolute units. The proposed reference intervals in RBC may be a useful tool when studying associations between fatty acids and health outcomes.

## Figures and Tables

**Table 1:** Background characteristics of the population.

Maternal age (years)	30.1 ± 4.6 *
Gestation (weeks)	16–32
Median (weeks)	28
Range (weeks)	17–40
	%
Body mass index (BMI **) in kg/m^2^	
<18.5	3.5
18.5–24.9	68.8
≥25	27.7
**Educational level**	
<4 years of higher education ^†^	60.7
≥4 years of higher education	39.3
**Marital status**	
Living with partner/married	96.8
Not living with partner/other	3.2
**Use of smoke/snuff tobacco during pregnancy**	
Yes	6.5
No	93.5
**Percentage of population per region**	
Northern Norway	13.8
Mid Norway	31.6
Western Norway	30.4
Eastern Norway	24.3

* mean ± standard deviation; ** BMI estimated for *n* = 202; ^†^ University or University College.

**Table 2:** Intake frequencies for seafood as dinner, seafood as spread/snack and omega-3 (ω-3) supplements among Norwegian pregnant women (*n* = 203). Unbracketed and bracketed figures represent the actual number of participants and the corresponding percentage (%).

	Assigned Score for PCA *		ω-3-Supplement Intake Distribution	
Seafood as dinner			Yes	No
<1time/month	1	9 (4.4)	5 (2.5)	4 (2.0)
1–3 times/month	2	35 (17.2)	27 (13.3)	8 (3.9)
1 time/week	3	96 (47.3)	74 (36.5)	22 (10.8)
2–3 times/week	4	59 (29.1)	46 (22.7)	13 (6.4)
≥4 times/week	5	4 (2)	4 (2.9)	
**Seafood as spread or snack**				
Never	1	24 (11.8)	15 (7.4)	9 (4.4)
Rare	2	45 (22,2)	35 (17.2)	10 (4.9)
1–3 times/month	3	56 (27.6)	41 (20.2)	15 (7.4)
1–2 times/week	4	59 (29.1)	50 (24.6)	9 (4.4)
3–5 times/week	5	17 (8.4)	15 (7.4)	2 (1.0)
≥5 times/week	6	2 (1)		2 (1.0)
**Total ω-3-supplement intake**	0 or 1		156 (76.9)	47 (23.2)

* PCA: principal component analysis.

**Table 3:** Percentiles for selected fatty acids in red blood cells from pregnant women (*n* = 247) were recruited all over Norway.

		Percentiles (%)									Percentiles (µg/g)									
	Mean	2.5	5	10	25	50	75	90	95	97.5	Mean	2.5	5	10	25	50	75	90	95	97.5
%																				
14:0	0.65	0.27	0.35	0.39	0.49	0.62	0.77	0.96	1.11	1.22	14.75	7.14	7.48	8.68	10.38	13.48	17.33	22.24	26.97	30.66
16:0	23.10	20.60	20.83	21.19	21.72	22.70	23.68	25.51	26.96	29.62	509.41	400.25	413.49	430.96	458.06	500.95	548.11	599.90	623.18	652.36
18:0	14.12	11.72	12.22	12.56	13.16	14.04	14.82	15.55	16.85	18.82	308.39	266.68	277.86	282.71	290.24	307.58	324.14	339.64	348.88	359.40
22:0	0.63	0.31	0.34	0.40	0.50	0.61	0.74	0.86	0.94	1.00	13.79	7.26	8.24	9.34	11.18	13.73	16.30	18.46	19.91	21.21
16:1	1.04	0.36	0.51	0.65	0.84	0.98	1.20	1.50	1.67	1.90	23.58	7.54	10.33	13.27	17.08	21.07	27.79	35.97	43.36	47.43
18:1	16.71	14.00	14.45	14.80	15.45	16.58	17.63	18.88	19.68	20.18	371.49	264.10	280.12	298.38	319.83	358.57	409.14	476.29	510.59	536.99
24:1ω-9	1.75	0.88	1.07	1.17	1.34	1.64	2.01	2.50	2.76	3.13	38.07	20.36	22.90	25.49	30.42	36.73	44.04	51.83	57.73	64.76
18:2ω-6	11.98	8.34	9.01	9.52	10.55	11.52	13.13	14.74	16.10	17.08	268.62	160.28	173.28	191.40	220.38	255.68	301.01	359.55	402.95	453.61
20:3ω-6	1.57	0.93	1.06	1.14	1.35	1.56	1.76	2.05	2.19	2.41	35.03	17.29	20.70	24.04	29.43	34.13	40.77	46.03	49.96	54.82
20:4ω-6	10.84	5.05	6.87	8.56	9.95	11.28	12.11	12.77	13.14	13.52	240.27	92.32	135.45	182.19	223.29	247.75	273.12	289.50	297.74	313.66
22:4ω-6	1.64	0.41	0.69	0.91	1.31	1.67	2.03	2.31	2.44	2.62	36.31	10.00	13.49	20.00	28.77	37.33	45.15	50.55	55.20	57.91
18:3ω-3	0.29	0.13	0.15	0.17	0.21	0.27	0.35	0.44	0.51	0.54	8.70	5.14	5.32	5.51	6.50	9.49	10.00	10.63	12.27	13.52
20:5ω-3	0.79	0.23	0.27	0.32	0.45	0.64	1.01	1.33	1.82	2.12	17.86	5.99	6.61	7.83	10.00	15.06	22.17	29.85	40.73	51.25
22:5ω-3	1.79	0.56	0.83	1.26	1.53	1.82	2.09	2.36	2.65	2.80	39.71	11.08	17.34	28.62	34.32	40.41	45.75	52.72	55.66	61.97
22:6ω-3	6.92	3.76	4.18	5.14	5.98	6.94	7.86	8.63	9.38	10.12	152.74	64.25	82.84	114.74	135.66	153.41	172.38	194.38	209.45	218.08
Omega-3 Index *	7.71	4.14	4.66	5.53	6.60	7.70	8.86	9.87	10.67	11.90										
ω6/ω3	2.71	1.63	1.85	1.97	2.29	2.65	3.03	3.54	3.76	3.93										
Total ω-6	26.65	17.83	21.01	23.44	25.62	27.16	28.52	29.74	30.34	30.85										
Total ω-3	10.24	5.68	6.28	7.85	8.89	10.32	11.67	12.66	13.33	15.14										

* Omega-3 Index is defined as the summation of 20:5ω-3 and 22:6ω-3.

**Table 4:** A comparison of reported mean [37,38,39,43] or median [40,41] concentrations (%) of fatty acids in different countries with those selected in the present study. The symbol × indicates that the fatty acid is not reported in the particular reference number. The green, yellow and red colors denote: Between 2.5 and 97.5 percentiles, under 2.5 percentile and over 97.5 percentile of the present study, respectively.

	Belgium [37]	Iceland [43]	Germany [39]	Japan [41]	Netherland [38]	Japan [40]
14:0		×			×	×
16:0		×			×	
18:0		×			×	
22:0	×	×	×		×	
16:1		×	×		×	×
18:1		×			×	
24:1ω-9	×	×	×		×	
18:2ω-6						
20:3ω-6			×			
20:4ω-6						
22:4ω-6	×					
18:3ω-3		×				×
20:5ω-3						
22:5ω-3	×					
22:6ω-3						

## Supplementary Materials

The following are available online at https://www.mdpi.com/2072-6643/12/10/2950/s1. Table S1: Fatty acid composition of the different brands of ω-3 supplements that were consumed by the participants. Table S2: Excel-based platform for generating automatically the normal distributions for the different fatty acids at the different health stations and the total fatty acid distributions. Table S3: Experimental e-FFQ (*n* = 203) and fatty acid (*n* = 247) results. The fatty acid results are expressed as relative (%) and absolute (mg/g RBC wet weight).

## Abbreviations

ALA	=	Alpha Linolenic Acid
DHA	=	DocosaHexaenoic Acid
e-FFQ	=	electronic-Food Frequency Questionnaire
EPA	=	EicosaPentaenoic Acid
FAME	=	Fatty Acid Methyl Esters
HUFA	=	Highly Unsaturated Fatty acids
LCPUFA	=	Long-Chain Polyunsaturated Fatty Acids
LiN	=	Little in Norway
PCA	=	Principal Component Analysis
PC1	=	Principal Component 1
PC2	=	Principal Component 2
PC3	=	Principal Component 3
PUFA	=	Polyunsaturated Fatty Acids
ω-3	=	Omega-3
ω-6	=	Omega-6
ω-9	=	Omega-9
RBC	=	Red Blood Cells
