# Supplementary material for: Fatty Acid Reference Intervals in Red Blood Cells among Pregnant Women in Norway–Cross Sectional Data from the ‘Little in Norway’ Cohort
Source: Nutrients. 2020 Sep 25;12(10):2950. doi: 10.3390/nu12102950 (PMC7601079; doi:10.3390/nu12102950)
Supplement: Supplementary file 1 [file nutrients-12-02950-s001.zip › TABLE S1.docx]

**Table S1:** Fatty acid composition of the different brands of ω-3 supplements that were consumed by the participants. The declared content per capsule by all the brands was 1 g of oil. The values are expressed in mg/g.

| Fatty acid | Supplement ***** | | | | | |
| --- | --- | --- | --- | --- | --- | --- |
|  | S_1_ | S_2_ | S_3_ | S_4_ | S_5_ | S_6_ |
| 14:0 | 2.55±0.07 | 22.20±0.00 | 1.25±0.07 | 3.05±0.07 | 2.55±0.07 | 1.95±1.06 |
| 15:0 | 0.00±0.00 | 1.45±0.07 | 0.00±0.00 | 0.00±0.00 | 0.00±0.00 | 0.00±0.00 |
| 16:0 | 22.30±0.14 | 58.65±0.21 | 24.05±0.21 | 22.70±0.00 | 12.50±0.14 | 19.00±5.23 |
| 17:0 | 0.00±0.00 | 0.00±0.00 | 0.00±0.00 | 0.00±0.00 | 0.00±0.00 | 2.40±0.42 |
| 18:0 | 29.80±0.14 | 19.00±0.28 | 31.55±0.07 | 26.75±0.35 | 14.40±0.28 | 25.95±5.44 |
| 20:0 | 3.35±0.07 | 2.40±0.14 | 4.80±0.00 | 9.25±0.07 | 4.30±0.00 | 5.00±2.26 |
| 22:0 | 0.95±0.07 | 0.85±0.07 | 0.00±0.00 | 2.40±0.00 | 0.00±0.00 | 0.60±0.85 |
| 24:0 | 0.00±0.00 | 1.15±0.07 | 0.00±0.00 | 0.00±0.00 | 0.00±0.00 | 0.00±0.00 |
| 16:1n-9 | 0.00±0.00 | 1.15±0.07 | 0.00±0.00 | 0.00±0.00 | 0.00±0.00 | 0.00±0.00 |
| 16:1n-7 | 8.60±0.14 | 26.10±0.28 | 8.45±0.21 | 8.40±0.00 | 7.30±0.14 | 8.30±0.57 |
| 18:1n-9 | 62.40±0.28 | 45.85±0.64 | 59.70±0.57 | 51.45±0.64 | 39.75±0.35 | 56.70±8.49 |
| 18:1n-7 | 18.35±0.07 | 14.20±0.42 | 21.70±0.28 | 20.05±0.21 | 13.70±0.00 | 17.60±1.56 |
| 20:1n-11 | 1.75±0.07 | 1.95±0.07 | 2.45±0.07 | 1.15±0.07 | 1.35±0.07 | 1.45±0.49 |
| 20:1n-9 | 13.50±0.00 | 13.80±0.14 | 24.00±0.28 | 23.10±0.14 | 19.35±0.21 | 18.80±6.93 |
| 20:1n-7 | 4.50±0.00 | 3.05±0.07 | 4.30±0.00 | 3.15±0.07 | 3.45±0.07 | 3.65±1.20 |
| 22:1n-11 | 11.40±0.28 | 20.00±0.85 | 15.40±0.14 | 8.70±0.28 | 10.75±0.07 | 9.85±2.33 |
| 22:1n-9 | 1.50±0.00 | 2.70±0.14 | 3.25±0.07 | 5.15±0.07 | 4.65±0.07 | 3.50±2.83 |
| 24:1n-9 | 9.35±0.07 | 5.30±0.42 | 11.35±0.07 | 12.15±0.07 | 16.75±0.64 | 13.20±1.84 |
| 16:2n-4 | 1.60±0.00 | 4.60±0.28 | 1.05±0.07 | 1.10±0.00 | 1.15±0.07 | 1.45±0.64 |
| 16:3n-3 | 2.35±0.07 | 6.10±0.14 | 1.55±0.07 | 1.65±0.07 | 1.45±0.07 | 1.60±0.57 |
| 18:2n-6 | 6.95±0.07 | 5.50±0.28 | 8.20±0.00 | 6.85±0.21 | 5.20±0.28 | 7.20±00 |
| 18:3n-3 | 3.70±0.00 | 3.40±0.14 | 5.75±0.07 | 4.50±0.00 | 3.20±0.14 | 3.80±0.14 |
| 18:4n-3 | 16.45±0.07 | 13.65±0.07 | 21.05±0.07 | 20.45±0.21 | 12.95±0.21 | 16.55±0.21 |
| 20:2n-6 | 2.10±0.00 | 1.60±0.00 | 2.55±0.07 | 2.15±0.07 | 2.15±0.07 | 2.10±0.14 |
| 20:3n-6 | 1.90±0.14 | 1.45±0.07 | 2.30±0.00 | 2.50±0.00 | 2.50±0.14 | 2.00±0.28 |
| 20:4n-6 | 13.00±0.14 | 9.15±0.35 | 17.65±0.07 | 15.55±0.21 | 15.10±0.28 | 13.45±0.78 |
| 20:4n-3 | 10.50±0.00 | 7.85±0.21 | 14.60±0.14 | 16.10±0.14 | 14.60±0.14 | 12.80±3.25 |
| 20:5n-3 | 243.80±1.70 | 174.55±0.49 | 282.65±1.63 | 274.75±2.33 | 255.20±7.21 | 238.95±1.20 |
| 22:5n-3 | 25.05±0.21 | 26.25±0.92 | 28.80±0.14 | 35.50±0.42 | 41.45±0.92 | 30.65±8.41 |
| 22:6n-3 | 168.70±0.99 | 175.10±10.32 | 174.35±1.06 | 174.15±1.63 | 190.75±11.1 | 167.60±6.79 |
| *****The commercial names of the different supplements (S_i_) are reported in reference [29] | | | | | | |
